# Prognostic value of the C-reactive protein-albumin-lymphocyte (CALLY) index in surgically treated non-small cell lung cancer

**DOI:** 10.3389/fnut.2026.1784875

**Published:** 2026-03-19

**Authors:** Kun Woo Kim, Hee Young Lee, Jae Ik Lee, Eung Chang Lee

**Affiliations:** 1 Department of Thoracic and Cardiovascular Surgery, Gil Medical Center, Gachon University College of Medicine, Incheon, Republic of Korea; 2 Department of Radiology, Gil Medical Center, Gachon University College of Medicine, Incheon, Republic of Korea; 3 International Healthcare Center, Seoul National University Bundang Hospital, Seongnam-si, Gyeonggi-do, Republic of Korea; 4 Department of General Surgery, Seongnam Citizens Medical Center, Seongnam-si, Gyeonggi-do, Republic of Korea

**Keywords:** CALLY index, non-small cell lung cancer, propensity score, survival analysis, thoracic surgery

## Abstract

**Background:**

The C-reactive protein-albumin-lymphocyte (CALLY) index is a novel composite biomarker integrating systemic inflammation, nutrition, and immunity. It has shown prognostic value in various malignancies. However, its role in non–small cell lung cancer (NSCLC), particularly in surgically treated patients, remains uncertain. Therefore, we evaluated the prognostic value of the CALLY index in comparison with established prognostic markers in patients with resected NSCLC.

**Methods:**

We retrospectively analyzed 680 patients who underwent curative-intent surgery for NSCLC. The independent prognostic value of the CALLY index was assessed using Cox proportional hazards models, and its discriminative performance was compared with established inflammatory, nutritional, and immune markers using time-dependent receiver operating characteristic analysis. The optimal cut-off value was determined by maximally selected log-rank statistics based on overall survival (OS). Propensity score matching (PSM) was applied to balance baseline characteristics. OS and recurrence-free survival (RFS) were evaluated using Kaplan–Meier analyses. Stratified Cox analyses and interaction tests were performed by pathological stage and smoking status to evaluate effect consistency of the CALLY index.

**Results:**

The CALLY index yielded the highest area under the curve (AUC = 0.675) for predicting OS among the evaluated markers, indicating relatively better prognostic performance. The optimal cut-off value was 5.14. In multivariable Cox analyses, a high CALLY index remained independently associated with improved overall survival [hazard ratio (HR) 0.40, 95% confidence interval (CI) 0.26–0.64; *p* < 0.001]. After 1:1 PSM, 208 matched pairs (*n* = 416) were generated with well-balanced clinical characteristics. Patients with a high CALLY index (>5.14) had significantly longer OS than those with a low index (≤5.14) (log-rank *p* < 0.001). However, RFS did not differ significantly between two groups (*p* = 0.701). The associations between the CALLY index and survival outcomes were consistent across pathological stage and smoking status.

**Conclusion:**

The preoperative CALLY index is an independent and accessible prognostic biomarker for OS in resected NSCLC. It may serve as a practical tool for risk stratification, guiding postoperative surveillance and adjuvant treatment planning.

## 1 Introduction

Lung cancer is a leading cause of cancer-related mortality worldwide, with non–small cell lung cancer (NSCLC) representing its most common subtype (1, 2). Despite improvements in screening and treatment, the prognosis of NSCLC remains heterogeneous, even among cases with similar clinical and pathological stages (3, 4). Surgical resection continues to be the cornerstone of curative therapy for early-stage NSCLC. However, considerable variability in recurrence rates and long-term survival persists. Such heterogeneity highlights the need for reliable, accessible, and integrative prognostic markers to refine postoperative risk stratification and personalize follow-up strategies.

Growing evidence suggests that the systemic host response—encompassing inflammation, nutritional reserves, and immune competence—plays a pivotal role in tumor progression, treatment response, and long-term outcomes across various malignancies, including NSCLC (5). Blood-based biomarkers reflecting these domains have gained attention as simple yet informative prognostic tools. Among them, the neutrophil-to-lymphocyte ratio (NLR) and platelet-to-lymphocyte ratio (PLR) have been extensively studied and shown prognostic value in NSCLC (6). However, the predictive performance of these individual biomarkers often varies across studies and may not fully encompass the complex interactions between the host factors and tumor biology.

To overcome the shortcomings of single-domain markers, recent studies have proposed composite markers integrating multiple physiological factors. The C-reactive protein-albumin-lymphocyte (CALLY) index is a novel composite biomarker proposed to reflect systemic inflammation [C-reactive protein (CRP)], nutritional status (serum albumin), and immunity (lymphocyte count). Initially developed and validated in patients with hepatocellular carcinoma, the CALLY index has since demonstrated prognostic value in gastrointestinal and hepatobiliary malignancies, supporting its broader oncologic applicability (7–12). Its ease of calculation from standard laboratory parameters further enhances feasibility for clinical practice.

Despite increasing application in other cancers, the prognostic value of the CALLY index remains underexplored in NSCLC, particularly in surgically treated patients where accurate risk stratification is crucial for postoperative decision-making. Moreover, its performance relative to other established inflammation-, nutrition-, and immunity-based prognostic biomarkers in this population has not been systematically assessed. Addressing this knowledge gap may not only validate the prognostic utility of the CALLY index in NSCLC but also refine current prognostic approaches.

Therefore, the aim of this study was to evaluate the prognostic significance of the CALLY index in surgically treated NSCLC. Specifically, we compared its predictive value for recurrence-free survival (RFS) and overall survival (OS) with other established systemic biomarkers, to assess its prognostic performance and clinical utility as a tool for postoperative risk stratification.

## 2 Methods

### 2.1 Patients

This retrospective, single-center cohort study was conducted at a university-affiliated tertiary hospital in South Korea. Between January 1, 2011 and December 31, 2020, a total of 745 patients with histologically confirmed NSCLC underwent surgery, including wedge resection, segmentectomy, lobectomy, and pneumonectomy. All surgeries were conducted by board-certified thoracic surgeons according to standardized institutional protocols.

Of the 745 patients, 65 were excluded due to the following reasons: (1) stage IV disease, (2) receipt of neoadjuvant therapy prior to curative-intent surgery, (3) history of another cancer within the preceding 5 years, (4) incomplete follow-up, or (5) missing laboratory data required for biomarker calculation. After applying these criteria, 680 patients who underwent curative-intent resection with complete perioperative records were included in the analysis.

Postoperatively, patients underwent scheduled follow-up with physical examination, routine blood tests, and chest computed tomography (CT) for every 4 months for the first 2 years and every 6 months thereafter. If recurrence was suspected, additional investigations, such as brain magnetic resonance imaging, abdominal CT, or positron emission tomography were undertaken. The recurrence date was determined as the day of pathological confirmation or, for clinically diagnosed cases, the day recurrence was first recognized by the attending physician.

### 2.2 Data collection and variables

Collected demographic and clinical variables included age, sex, body mass index (BMI), smoking history, chronic obstructive pulmonary disease (COPD) status, American Society of Anesthesiologists (ASA) score, type of surgery, tumor histologic subtype, Tumor–Node–Metastasis (TNM) stage according to the 8th edition of the American Joint Committee on Cancer (AJCC), and use of neoadjuvant or adjuvant therapy. Histological classification of lung cancer was based on criteria proposed by the International Association for the Study of Lung Cancer, the American Thoracic Society, and the European Respiratory Society (13).

### 2.3 Calculation of biomarkers

The CALLY index was defined as follows (7, 8):

CALLY = (Albumin [g/dL] × Lymphocyte count [/mm^3^]) / (CRP [mg/dL] × 10^4^).

In addition to the CALLY index, we computed other established systemic inflammation and nutrition markers, including the NLR, PLR, systemic immune-inflammation index (SII), prognostic nutritional index (PNI), hemoglobin, albumin, lymphocyte, and platelet (HALP) score, Glasgow Prognostic Score (GPS), modified GPS (mGPS), albumin-to-alkaline phosphatase ratio (AAPR), and advanced lung cancer inflammation index (ALI), using definitions (Supplementary Table S1) from previous studies (14–20). Laboratory data for biomarker analysis were obtained from blood samples collected within 1 month before surgery.

### 2.4 Statistical analysis

Group comparisons were performed using chi-square or Fisher’s exact tests for categorical variables and Student’s *t*-test or Mann–Whitney *U* test for continuous variables, as appropriate to data distribution. To evaluate the discriminative ability of the CALLY index and other systemic inflammation, immunity, and nutrition markers, time-dependent receiver operating characteristic (ROC) curves were generated, and the area under the curve (AUC) values were calculated using the timeROC package in R software. The optimal cut-off of the CALLY index was determined with the maximally selected rank statistics method (the surv_cutpoint function in the survminer package), which identifies the most significant split point for survival stratification—in this case, 5-year OS—based on log-rank statistics. Multivariable Cox proportional hazards models were performed in the entire cohort to assess the association between the CALLY index and OS and RFS, with adjustment for clinically relevant covariates.

To minimize baseline imbalances between comparison groups and to facilitate a clinically interpretable comparison of survival outcomes, we conducted 1:1 nearest-neighbor propensity score matching (PSM) without replacement. Propensity scores were estimated using multivariable logistic regression including age, sex, BMI, smoking history, COPD, ASA class, tumor histology, pathological stage, surgical procedure, and perioperative treatment. A caliper width of 0.05 was applied to ensure matching quality and limit residual bias. OS and RFS were analyzed with the Kaplan–Meier method, and intergroup survival differences were assessed through the log-rank test.

Further, given the biological and clinical heterogeneity of lung cancer, we evaluated whether the prognostic association of the CALLY index was consistent across key disease- and patient-related characteristics, most notably pathological stage and smoking status. Accordingly, stratified Cox proportional hazards models were constructed according to pathological stage and smoking status (never vs. ever smokers), and potential effect modification was examined by incorporating corresponding interaction terms, with statistical significance assessed using likelihood ratio tests. Subgroup-specific hazard ratios (HRs) with 95% confidence intervals (CIs) were summarized in forest plots.

All statistical analyses were two-sided, with *p*-values < 0.05 considered statistically significant. Statistical analyses were carried out with R software (version 4.5.1; R Foundation for Statistical Computing, Vienna, Austria).

### 2.5 Ethical considerations

This study was approved by the Institutional Review Board (GBIRB2025-243). Informed consent was waived owing to its retrospective design. All data were de-identified and managed in accordance with institutional guidelines and the Declaration of Helsinki for research involving human participants.

## 3 Results

### 3.1 Patient characteristics

A total of 680 patients with NSCLC who underwent curative-intent resection were analyzed. The median age was 66 years [interquartile range (IQR): 59–73], and 59.4% were male (*n* = 404). BMI distribution showed 4.9% underweight, 34.9% normal, 36.0% overweight, and 24.3% obese.

Regarding smoking status, 30.4% were current smokers, 29.1% ex-smokers, and 40.4% never smokers. COPD was present in 35.7% of patients. Most were ASA class 2 (90.6%), followed by class 3 (5.9%), class 1 (3.2%), and class 4 (0.3%). Lobectomy was the most common procedure (81.5%), followed by wedge resection (13.5%), pneumonectomy (2.4%), and segmentectomy (2.6%). Histologically, adenocarcinoma predominated (70.3%), with squamous cell carcinoma (23.7%), adenosquamous carcinoma (2.1%), and others (4.0%) comprising the remainder.

By AJCC 8th edition staging, 63.7% were stage I or carcinoma *in situ* (Tis), 21.3% stage II, and 15.0% stage III. Postoperatively, 61.6% received no adjuvant therapy, 32.1% received chemotherapy, 5.4% chemoradiation, and 0.9% radiotherapy alone. The median preoperative CALLY index was 8.1 (IQR: 2.3–18.8). Table 1 summarizes these clinical, pathological, and treatment-related characteristics.

**Table 1:** Patients characteristics.

Variables	*N* = 680
Age (yr)	66 (59–73)
Sex	
Female	276 (40.6%)
Male	404 (59.4%)
BMI^a^ (kg/m^2^)	
Under weight (<18.5)	33 (4.9%)
Normal (≥18.5, <23)	237 (34.9%)
Overweight (≥23, <25)	245 (36.0%)
Obese (≥ 25)	165 (24.3%)
Smoking	
Current	207 (30.4%)
Ex-smoker	198 (29.1%)
Never-smoker	275 (40.4%)
COPD	
No	437 (64.3%)
Yes	243 (35.7%)
ASA status	
1	22 (3.2%)
2	616 (90.6%)
3	40 (5.9%)
4	2 (0.3%)
Operation name	
Wedge resection	92 (13.5%)
Segmentectomy	18 (2.6%)
Lobectomy	554 (81.5%)
Pneumonectomy	16 (2.4%)
Histological type	
Adenocarcinoma	478 (70.3%)
Squamous cell carcinoma	161 (23.7%)
Adenosquamous carcinoma	14 (2.1%)
Others	27 (4.0%)
Stage (AJCC 8th)	
I, Tis	433 (63.7%)
II	145 (21.3%)
III	102 (15.0%)
Adjuvant treatment	
None	419 (61.6%)
Chemotherapy	218 (32.1%)
Concurrent chemoradiation therapy	37 (5.4%)
Radiotherapy	6 (0.9%)
CALLY index	8.1 (2.3–18.8)

a BMI values were categorized as underweight (less than 18.5 kg/m^2^), normal (18.5–22.9 kg/m^2^), overweight (23.0–24.9 kg/m^2^), or obese (greater than or equal to 25 kg/m^2^).

Data are presented as number (%) or median (interquartile range).

AJCC, American Joint Committee on Cancer; ASA, American Society of Anesthesiologists; BMI, body mass index; CALLY, C-reactive protein-albumin-lymphocyte; COPD, chronic obstructive pulmonary disease; Tis, carcinoma *in situ*.

### 3.2 Comparison of CALLY index with other composite biomarkers

The prognostic performance of the CALLY index was evaluated through time-dependent ROC analyses for 5-year RFS and OS, and compared with established markers of inflammation, nutrition, and immunity.

**Figure 1:**
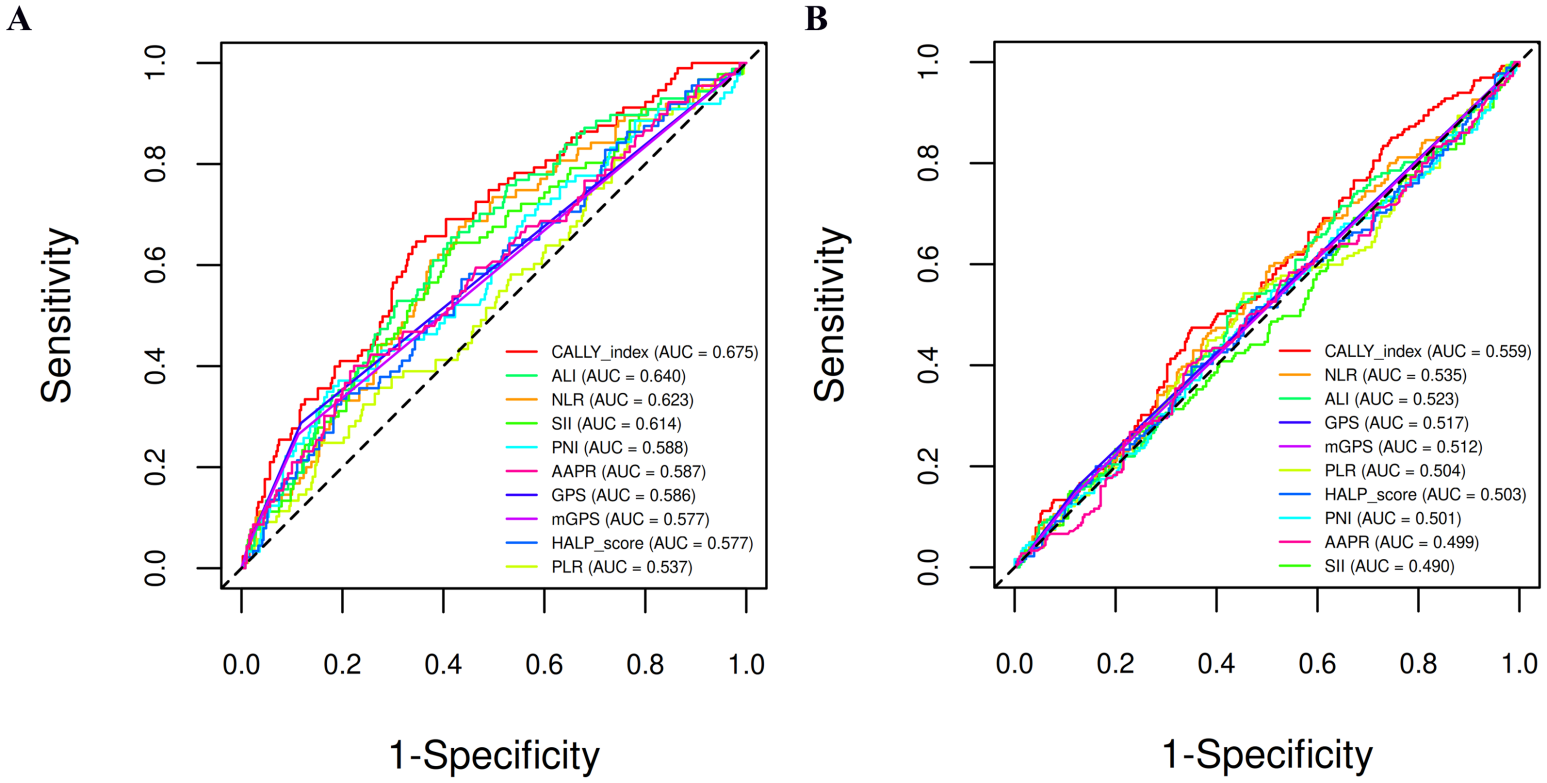
Time-dependent ROC curves at 5 years for overall survival **(A)** and recurrence-free survival **(B)**, comparing the CALLY index with other composite biomarkers that integrate systemic inflammation, nutritional status, and immune function. AAPR, albumin-to-alkaline phosphatase ratio; ALI, advanced lung cancer inflammation index; AUC, area under the curve; CALLY, C-reactive protein-albumin-lymphocyte; GPS, Glasgow Prognostic Score; HALP, hemoglobin, albumin, lymphocyte, and platelet; mGPS, modified Glasgow Prognostic Score; NLR, neutrophil-to-lymphocyte ratio; PLR, platelet-to-lymphocyte ratio; PNI, prognostic nutritional index; ROC, receiver operating characteristic; SII, systemic immune-inflammation index.

For OS, the CALLY index showed the highest AUC of 0.675 [95% confidence interval (CI): 0.614–0.736], outperforming the ALI (AUC = 0.640), NLR (AUC = 0.623), and SII (AUC = 0.614). Other indices—including the GPS, mGPS, PLR, HALP score, PNI, and AAPR—had lower or comparable performance (Table 2 and Figure 1A).

**Table 2:** Comparative analysis of 5-year time-dependent ROC curves of the CALLY index versus other systemic inflammation–nutrition–immunity markers for postoperative prediction of RFS and OS.

Markers	AUC	OS	*p*	AUC	RFS	*p*
		95% CI			95% CI	
CALLY index	0.675	0.614–0.736	—	0.559	0.506–0.611	—
ALI	0.640	0.577–0.703	0.433	0.523	0.470–0.576	0.353
NLR	0.623	0.560–0.687	0.250	0.535	0.481–0.588	0.530
SII	0.614	0.548–0.679	0.179	0.49	0.436–0.545	0.076
PNI	0.588	0.520–0.656	0.062	0.501	0.448–0.555	0.135
AAPR	0.587	0.519–0.654	0.057	0.499	0.446–0.553	0.120
GPS	0.586	0.536–0.635	0.026	0.517	0.485–0.550	0.191
mGPS	0.577	0.528–0.626	0.014	0.512	0.481–0.544	0.138
HALP score	0.577	0.510–0.643	0.033	0.503	0.449–0.557	0.150
PLR	0.537	0.470–0.603	0.003	0.504	0.450–0.558	0.156

AAPR, albumin-to-alkaline phosphatase ratio; ALI, advanced lung cancer inflammation index; AUC, area under the curve; CALLY, C-reactive protein-albumin-lymphocyte; CI, confidence interval; GPS, Glasgow Prognostic Score; HALP, hemoglobin, albumin, lymphocyte, and platelet; mGPS, modified Glasgow Prognostic Score; NLR, neutrophil-to-lymphocyte ratio; OS, overall survival; PLR, platelet-to-lymphocyte ratio; PNI, prognostic nutritional index; RFS, recurrence-free survival; ROC, receiver operating characteristic; SII, systemic immune inflammation index.

In contrast, for 5-year RFS prediction, the prognostic capacity of all tested markers was attenuated (Figure 1B). The CALLY index yielded an AUC of 0.559 (95% CI: 0.506–0.611), remaining the highest among them but without strong discrimination. This indicates that such biomarkers are more predictive of OS than RFS, likely reflecting their closer association with host resilience and treatment tolerance rather than tumor biology.

Overall, these findings suggest that, among the evaluated systemic indices, the CALLY index may be the most informative for preoperative prediction of long-term mortality in NSCLC, although its absolute discriminative performance remains modest.

### 3.3 Univariate and multivariate Cox proportional hazards analyses

In univariate Cox proportional hazards analyses, a high CALLY index was significantly associated with improved OS [hazard ratio (HR) 0.32, 95% confidence interval (CI) 0.21–0.50; *p* < 0.001] and RFS (HR 0.68, 95% CI 0.51–0.92; *p* = 0.011). In the multivariable Cox model for OS, older age (HR 1.06 per year, 95% CI 1.03–1.09; *p* < 0.001) and male sex (HR 2.59, 95% CI 1.47–4.59; *p* = 0.001) were independently associated with poorer survival. Among multi-level variables, body mass index (*p* = 0.021) and pathological stage (*p* < 0.001) were also significant prognostic factors. Conversely, a high CALLY index was independently associated with improved survival (HR 0.40, 95% CI 0.26–0.64; *p* < 0.001). In the multivariable analysis for RFS, older age (HR 1.02 per year, 95% CI 1.01–1.04; *p* = 0.007) and chronic obstructive pulmonary disease (HR 1.54, 95% CI 1.13–2.10; *p* = 0.006) were independently associated with higher recurrence risk. Pathological stage (*p* < 0.001) and receipt of adjuvant treatment (*p* = 0.039) also remained significant. However, the CALLY index did not retain independent prognostic significance for RFS after adjustment. Detailed results are presented in Tables 3, 4.

**Table 3:** Univariate and multivariate Cox proportional hazards analyses of OS.

Variable	Univariate analysis		Multivariate analysis	
	HR (95% CI)	*p*	HR (95% CI)	*p*
Age^a^ (yr)	1.05 (1.03–1.08)	<0.001	1.06 (1.03–1.09)	<0.001
Sex^b^ (female vs. male)	3.42 (1.99–5.87)	<0.001	2.59 (1.47–4.59)	0.001
BMI^c^ (kg/m^2^)		0.073		0.021
Under weight (<18.5)	Reference		Reference	
Normal (≥18.5, <23)	0.49 (0.20–1.20)	0.120	0.47 (0.19–1.17)	0.105
Overweight (≥23, <25)	0.83 (0.35–1.96)	0.675	0.91 (0.38–2.19)	0.841
Obese (≥ 25)	0.49 (0.19–1.23)	0.129	0.46 (0.18–1.19)	0.112
Smoking		0.002		
Current	Reference			
Ex-smoker	0.88 (0.55–1.42)	0.601		
Never-smoker	0.40 (0.23–0.68)	<0.001		
COPD^b^ (no vs. yes)	1.45 (0.95–2.20)	0.082	0.71 (0.45–1.11)	0.132
ASA status		0.937		
1	Reference			
2	1.58 (0.39–6.41)	0.525		
3	1.64 (0.32–8.46)	0.554		
4	NE^d^	0.995		
Operation name		0.089		
Wedge resection	Reference			
Segmentectomy	3.07 (1.03–9.16)	0.044		
Lobectomy	1.29 (0.64–2.57)	0.476		
Pneumonectomy	2.82 (0.87–9.16)	0.084		
Histological type		0.001		
Adenocarcinoma	Reference			
Squamous cell carcinoma	2.09 (1.33–3.28)	0.001		
Adenosquamous carcinoma	3.50 (1.26–9.71)	0.016		
Others	2.50 (1.07–5.85)	0.034		
Stage (AJCC 8th)		<0.001		<0.001
I, Tis	Reference		Reference	
II	2.48 (1.50–4.10)	<0.001	1.91 (1.14–3.20)	0.013
III	3.78 (2.28–6.24)	<0.001	3.88 (2.31–6.54)	<0.001
Adjuvant treatment		<0.001		
None	Reference			
Chemotherapy	2.50 (1.61–3.89)	<0.001		
Concurrent chemoradiation therapy	2.69 (1.25–5.78)	0.012		
Radiotherapy	2.18 (0.30–15.91)	0.442		
CALLY index^b^ (≤5.14 vs. >5.14)	0.32 (0.21–0.50)	<0.001	0.40 (0.26–0.64)	<0.001

a The HR per 1-year increase in age.

b The value on the left is the reference.

c BMI values were categorized as underweight (less than 18.5 kg/m^2^), normal (18.5–22.9 kg/m^2^), overweight (23.0–24.9 kg/m^2^), or obese (greater than or equal to 25 kg/m^2^).

d Not estimable due to absence of events in this subgroup.

AJCC, American Joint Committee on Cancer; ASA, American Society of Anesthesiologists; BMI, body mass index; CALLY, C-reactive protein-albumin-lymphocyte; COPD, chronic obstructive pulmonary disease; HR, hazard ratio; OS, overall survival; Tis, carcinoma *in situ*.

**Table 4:** Univariate and multivariate Cox proportional hazards analyses of RFS.

Variable	Univariate analysis		Multivariate analysis	
	HR (95% CI)	*p*	HR (95% CI)	*p*
Age^a^ (yr)	1.02 (1.00–1.03)	0.050	1.02 (1.01–1.04)	0.007
Sex^b^ (female vs. male)	1.39 (1.03–1.89)	0.034		
BMI^c^ (kg/m^2^)		0.920		
Under weight (<18.5)	Reference			
Normal (≥18.5, <23)	1.08 (0.49–2.35)	0.856		
Overweight (≥23, <25)	1.14 (0.52–2.49)	0.737		
Obese (≥25)	1.21 (0.55–2.68)	0.633		
Smoking		0.044		
Current	Reference			
Ex-smoker	1.00 (0.69–1.43)	0.983		
Never-smoker	0.67 (0.47–0.96)	0.029		
COPD^b^ (no vs. yes)	1.60 (1.19–2.14)	0.002	1.54 (1.13–2.10)	0.006
ASA status		0.273		
1	Reference			
2	2.40 (0.77–7.50)	0.134		
3	1.39 (0.35–5.54)	0.645		
4	NE^d^	0.992		
Operation name		0.575		
Wedge resection	Reference			
Segmentectomy	1.84 (0.78–4.35)	0.166		
Lobectomy	1.24 (0.77–1.97)	0.375		
Pneumonectomy	1.22 (0.42–3.57)	0.716		
Histological type		0.333		
Adenocarcinoma	Reference			
Squamous cell carcinoma	1.26 (0.90–1.78)	0.185		
Adenosquamous carcinoma	1.79 (0.73–4.38)	0.202		
Others	1.33 (0.65–2.73)	0.430		
Stage (AJCC 8th)		<0.001		<0.001
I, Tis	Reference		Reference	
II	1.79 (1.22–2.62)	0.003	1.07 (0.64–1.78)	0.796
III	5.50 (3.92–7.73)	<0.001	3.45 (2.06–5.78)	<0.001
Adjuvant treatment		<0.001		0.039
None	Reference		Reference	
Chemotherapy	2.71 (1.97–3.73)	<0.001	1.92 (1.18–3.11)	0.008
Concurrent chemoradiation therapy	5.51 (3.41–8.91)	<0.001	2.14 (1.11–4.13)	0.023
Radiotherapy	4.58 (1.44–14.56)	0.010	2.66 (0.80–8.81)	0.110
CALLY index^b^ (≤5.14 vs. >5.14)	0.68 (0.51–0.92)	0.011		

a The HR per 1-year increase in age.

b The value on the left is the reference.

c BMI values were categorized as underweight (less than 18.5 kg/m^2^), normal (18.5–22.9 kg/m^2^), overweight (23.0–24.9 kg/m^2^), or obese (greater than or equal to 25 kg/m^2^).

d Not estimable due to absence of events in this subgroup.

AJCC, American Joint Committee on Cancer; ASA, American Society of Anesthesiologists; BMI, body mass index; CALLY, C-reactive protein-albumin-lymphocyte; COPD, chronic obstructive pulmonary disease; HR, hazard ratio; RFS, recurrence-free survival; Tis, carcinoma *in situ*.

### 3.4 CALLY-based risk group classification and PSM

**Figure 2:**
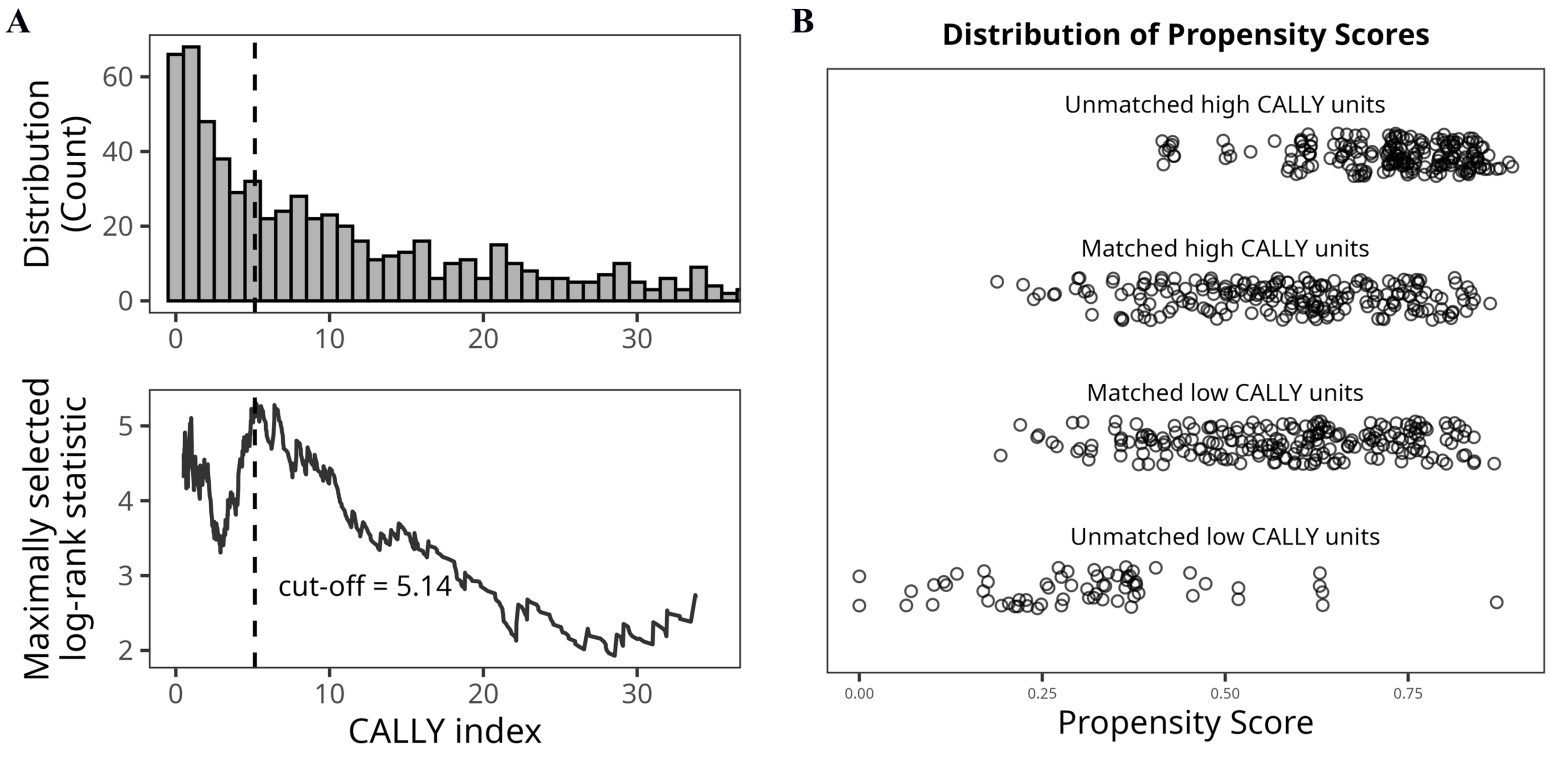
**(A)** Distribution of the CALLY index and determination of the optimal cut-off for 5-year overall survival based on maximally selected log-rank statistics (maxstat method). **(B)** Distribution of propensity scores before and after 1:1 matching between the high and low CALLY index groups. Matched and unmatched treated/control units are separately displayed, confirming adequate overlap and balance after matching. CALLY, C-reactive protein-albumin-lymphocyte.

Among 680 patients who underwent curative-intent resection for NSCLC, 407 (59.9%) were classified into the high CALLY index group (>5.14) and 273 (40.1%) into the low group (≤5.14), based on the optimal cut-off from maximally selected rank statistics (Figure 2A).

To minimize baseline differences between groups, 1:1 nearest-neighbor PSM was performed without replacement, using a caliper width of 0.05, yielding 208 matched pairs (*n* = 416). After matching, excellent covariate balance was achieved, with standardized mean differences < 0.1 for all variables and substantial overlap observed in the propensity score distributions between groups (Figure 2B). Matched baseline characteristics are summarized in Table 5, with no significant differences between groups across key clinical and pathological variables.

**Table 5:** Comparison of baseline data stratified by CALLY index after PSM.

Variables	Total (*N* = 416)	Low (*N* = 208)	High (*N* = 208)	*p*	Test SMD
Age	66 (59–73)	66 (59–72)	66 (59–73)	0.725	0.030
Sex				0.611	0.060
Female	152 (36.5%)	73 (35.1%)	79 (38.0%)		
Male	264 (63.5%)	135 (64.9%)	129 (62.0%)		
BMI^a^ (kg/m^2^)				0.921	0.069
Under weight (<18.5)	20 (4.8%)	10 (4.8%)	10 (4.8%)		
Normal (≥18.5, <23)	134 (32.2%)	70 (33.7%)	64 (30.8%)		
Overweight (≥23, <25)	145 (34.9%)	72 (34.6%)	73 (35.1%)		
Obese (≥25)	117 (28.1%)	56 (26.9%)	61 (29.3%)		
Smoking				0.824	0.061
Current	142 (34.1%)	73 (35.1%)	69 (33.2%)		
Ex-smoker	126 (30.3%)	64 (30.8%)	62 (29.8%)		
Never-smoker	148 (35.6%)	71 (34.1%)	77 (37.0%)		
COPD				0.764	0.039
No	250 (60.1%)	127 (61.1%)	123 (59.1%)		
Yes	166 (39.9%)	81 (38.9%)	85 (40.9%)		
ASA status				0.717	0.080
1	8 (1.9%)	5 (2.4%)	3 (1.4%)		
2	384 (92.3%)	192 (92.3%)	192 (92.3%)		
3	24 (5.8%)	11 (5.3%)	13 (6.2%)		
Operation name				0.937	0.063
Wedge resection	50 (12.0%)	24 (11.5%)	26 (12.5%)		
Segmentectomy	12 (2.9%)	7 (3.4%)	5 (2.4%)		
Lobectomy	350 (84.1%)	175 (84.1%)	175 (84.1%)		
Pneumonectomy	4 (1.0%)	2 (1.0%)	2 (1.0%)		
Histological type				0.944	0.060
Adenocarcinoma	285 (68.5%)	142 (68.3%)	143 (68.8%)		
Squamous cell carcinoma	106 (25.5%)	53 (25.5%)	53 (25.5%)		
Adenosquamous carcinoma	11 (2.6%)	5 (2.4%)	6 (2.9%)		
Others	14 (3.4%)	8 (3.8%)	6 (2.9%)		
Stage (AJCC 8th)				0.861	0.054
I, Tis	254 (61.1%)	125 (60.1%)	129 (62.0%)		
II	94 (22.6%)	47 (22.6%)	47 (22.6%)		
III	68 (16.3%)	36 (17.3%)	32 (15.4%)		
Adjuvant treatment				0.997	0.021
None	240 (57.7%)	120 (57.7%)	120 (57.7%)		
Chemotherapy	147 (35.3%)	74 (35.6%)	73 (35.1%)		
Concurrent chemoradiation therapy	25 (6.0%)	12 (5.8%)	13 (6.2%)		
Radiotherapy	4 (1.0%)	2 (1.0%)	2 (1.0%)		

Data are presented as number (%) or median (interquartile range).

a BMI values were categorized as underweight (less than 18.5 kg/m^2^), normal (18.5–22.9 kg/m^2^), overweight (23.0–24.9 kg/m^2^), or obese (greater than or equal to 25 kg/m^2^).

AJCC, American Joint Committee on Cancer; ASA, American Society of Anesthesiologists; BMI, body mass index; CALLY, C-reactive protein-albumin-lymphocyte; COPD, chronic obstructive pulmonary disease; PSM, propensity score matching; SMD, standardized mean difference; Tis, carcinoma *in situ*.

This well-balanced post-matching cohort preserved the representativeness of the original population while enabling more valid estimation of the prognostic significance of the CALLY index for survival outcomes.

### 3.5 Survival outcomes after PSM

**Figure 3:**
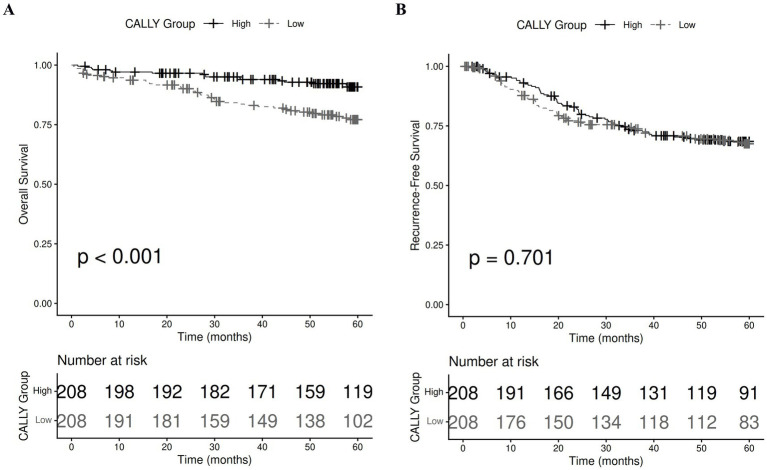
Kaplan–Meier curves comparing low versus high CALLY index groups for overall survival **(A)** and recurrence-free survival **(B)** after PSM. CALLY, C-reactive protein-albumin-lymphocyte; PSM, propensity score matching.

Within the propensity score-matched cohort, Kaplan–Meier curves revealed a significant difference in OS by CALLY index stratification. Patients in the high CALLY index group had better long-term survival, with curves diverging early and remaining distinctly separated over the 5-year follow-up period (log-rank *p* < 0.001, Figure 3A).

Conversely, no statistically significant difference in RFS was observed between the two groups; Kaplan–Meier curves were largely superimposed throughout the follow-up period (log-rank *p* = 0.701, Figure 3B).

These results suggest that the CALLY index, by integrating systemic inflammation, nutritional status, and immune competence, is associated with OS but not RFS in surgically treated NSCLC.

### 3.6 Stratified and interaction analyses of the CALLY index by pathological stage and smoking status

**Figure 4:**
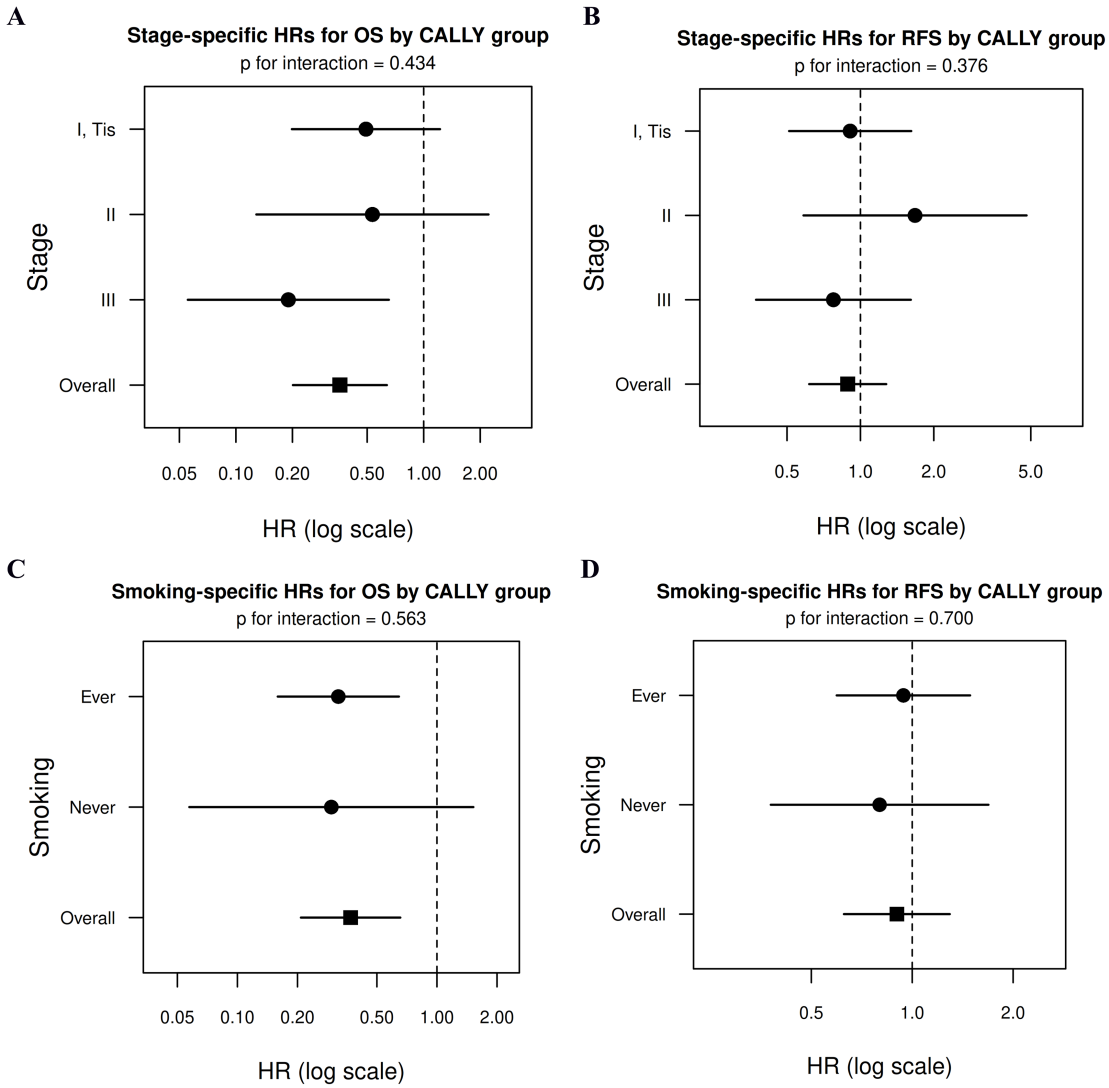
Forest plots of subgroup-specific hazard ratios (HRs). Stage-specific analyses for **(A)** overall survival (OS) and **(B)** recurrence-free survival (RFS), and smoking status-specific analyses for **(C)** OS and **(D)** RFS, according to CALLY group. The square denotes the overall HR derived from stratified Cox proportional hazards models. Interaction *p*-values were calculated using likelihood ratio tests, and no significant interaction was observed between the CALLY index and pathological stage or smoking status for either OS or RFS. CALLY, C-reactive protein-albumin-lymphocyte.

When pathological stage was treated as a stratification factor in the Cox model, a high CALLY index remained significantly associated with improved OS (HR = 0.358, 95% CI 0.201–0.637; *p* < 0.001), with no evidence of effect modification by stage (*p* for interaction = 0.434; Figure 4A). In contrast, no significant association between the CALLY index and RFS was observed in the stage-stratified analysis (HR = 0.887, 95% CI 0.616–1.276; *p* = 0.518), and interaction testing likewise did not indicate effect modification by stage (*p* for interaction = 0.376, Figure 4B).

Consistent findings were observed in analyses accounting for smoking status. After stratification by smoking status, a high CALLY index remained significantly associated with improved OS (HR = 0.369, 95% CI 0.208–0.655; *p* < 0.001), and formal interaction testing did not indicate effect modification by smoking status (*p* for interaction = 0.563; Figure 4C). Similarly, no significant association with RFS was observed after stratification by smoking status (HR = 0.900, 95% CI 0.625–1.294; *p* = 0.569), and no evidence of effect modification was detected (*p* for interaction = 0.700; Figure 4D).

Taken together, these analyses indicate that the prognostic association of the CALLY index with OS, as well as its lack of association with RFS, was consistent across pathological stage and smoking status, with no evidence of interaction-based effect modification for either outcome.

## 4 Discussion

The CALLY index integrates three routinely available and physiologically informative parameters: CRP, serum albumin, and peripheral lymphocyte count. Each component reflects a distinct yet interrelated dimension of the host’s systemic state. CRP is a prototypical acute-phase reactant that captures the magnitude of systemic inflammation and is regulated by pro-inflammatory cytokines such as interleukin-6 (IL-6). Elevated CRP levels have been consistently associated with worse oncologic outcomes across multiple types of cancer, including NSCLC (21–24). Serum albumin indexes nutritional status and functions as a negative acute-phase reactant; hypoalbuminemia commonly accompanies cachexia, catabolism, or advanced disease. Lymphocyte count is a surrogate for immune competence and plays a central role in anti-tumor immune surveillance. The CALLY index integrates these three domains to deliver a holistic assessment of systemic inflammation, nutrition, and immunity, all of which are crucial determinants of cancer prognosis. Markers such as the NLR primarily reflect immune status, whereas the PNI combines nutritional and immune components but has limited sensitivity to inflammation. By simultaneously representing inflammatory, nutritional, and immune signals, the CALLY index enables a more precise assessment of the patient’s physiological status.

In this retrospective cohort of 680 surgically treated NSCLC patients, the preoperative CALLY index emerged as a clinically useful and accessible predictor of long-term survival. Among systemic inflammation, nutrition, and immunity-related indices, the CALLY index showed superior prognostic accuracy for OS. Importantly, its prognostic significance persisted after rigorous adjustment through PSM, supporting robustness and potential clinical utility.

The CALLY index achieved the highest AUC for OS among all tested markers, indicating comparatively better discriminatory capacity than other indices. After PSM adjustment, patients with a high CALLY index experienced significantly better OS outcomes (*p* < 0.001), whereas no significant difference was observed in RFS (*p* = 0.701). This suggests that the index reflects overall systemic resilience or physiological reserve rather than tumor-specific recurrence risk. The fact that CALLY index influenced OS but not RFS is consistent with earlier studies in gastrointestinal and hepatobiliary cancers, where the index was shown to predict treatment tolerance, treatment related complications and non-cancer mortality (12, 25). These findings highlight the importance of incorporating host-related factors into prognostic models, particularly in surgically treated populations where baseline physiological status may substantially influence postoperative recovery and long-term outcomes.

Importantly, in multivariable Cox proportional hazards models including pathological stage and smoking status, the CALLY index (HR 0.40, 95% CI 0.26–0.64; *p* < 0.001) remained an independent prognostic factor for OS in the entire cohort. This association was further supported by PSM, which demonstrated consistent survival differences between CALLY groups after balancing baseline clinical characteristics. Moreover, stratified and interaction analyses showed that the association between the CALLY index and OS was directionally consistent across pathological stage and smoking subgroups, with no statistically significant evidence of effect modification. Together, these findings suggest that the prognostic signal of the CALLY index is unlikely to reflect residual confounding driven by a specific stage category or smoking subgroup. Nevertheless, given the biological and clinical heterogeneity between smoking-related and never-smoking lung cancer, as well as across disease stages, these results should be interpreted as evidence of robustness and internal consistency within the surgically treated cohort rather than as proof of full interchangeability across fundamentally distinct populations.

Traditional staging systems such as TNM primarily emphasize tumor burden and anatomical extent but often overlook inter-individual differences in systemic health. Our observations add to evidence that integrating tumor- and host-related factors can enhance prognostic accuracy and inform personalized treatment strategies.

The biological plausibility of the CALLY index lies in the synergistic interaction of its components. Chronic inflammation, as captured by elevated CRP, is known to promote angiogenesis, DNA damage, epithelial-mesenchymal transition, and immune evasion—hallmarks of cancer progression (23, 26–29). Hypoalbuminemia is frequently associated with muscle wasting, impaired drug metabolism, and delayed wound healing, all of which may adversely affect cancer recovery and survival. Lymphopenia, indicative of diminished adaptive immunity, compromises anti-tumor cytotoxic responses and is associated with poor prognosis in various cancers. The ability of the CALLY index to combine these elements into a singular metric offers both conceptual clarity and practical relevance.

Clinically, the CALLY index may have several applications. Preoperatively, it enables the identification of high-risk patients who could benefit from closer surveillance, nutritional or immunologic optimization, and timely initiation of adjuvant therapy. The CALLY index is based on inexpensive, routine laboratory tests, making it highly scalable and adaptable even in resource-limited settings. Unlike more complex biomarkers such as genomic signatures or radiomics, it requires no specialized equipment or interpretation.

The CALLY index may also serve as a surrogate of physiological reserve, especially in older or comorbid patients. This is especially relevant in the era of minimally invasive thoracic surgery and enhanced recovery after surgery protocols, where precise risk assessment is essential for patient selection and perioperative planning. Future studies are needed to clarify whether the CALLY index can predict tolerance to systemic therapies, including chemotherapy, immunotherapy, or multimodal regimens, particularly in patients with borderline functional status.

Conventional tumor-centric prognostic models—such as TNM staging, maximum standardized uptake value, and histopathological features—primarily reflect the tumor’s overall burden, anatomical extent, and metabolic activity. However, they provide limited insight into systemic inflammation, immune competence, or nutritional state. Consequently, outcomes such as postoperative complications, recovery capacity, and non-cancer mortality are not adequately captured by tumor-based parameters alone. The CALLY index addresses this gap by incorporating patient-related factors, thereby complementing traditional tumor-based models and representing a potential paradigm shift in prognostic evaluation.

The simplicity of CALLY index calculation also supports its integration into digital platforms such as clinical decision support systems and electronic medical records. Automated calculation and flagging of high-risk patients based on predefined thresholds could assist clinicians in identifying patients who require additional evaluation or intervention. Embedding the index into validated risk calculators could further standardize preoperative assessments, strengthen multidisciplinary discussions, and improve consistency in patient care across institutions.

Evaluating temporal trends in the CALLY index is another key direction for future investigation. Monitoring how the CALLY index fluctuates before and after surgery or during systemic therapy may provide valuable dynamic insights into treatment response, postoperative recovery, or early signs of clinical deterioration. Such longitudinal monitoring could facilitate real-time adjustments to clinical strategies, enabling proactive interventions. For instance, a gradual decline in the postoperative CALLY index might indicate the need for enhanced nutritional or immunologic support to prevent complications or reduce mortality.

Integrating the CALLY index into multifactorial prognostic models also warrants exploration. As thoracic oncology moves toward precision medicine, there is increasing momentum to combine molecular, radiological, and clinical biomarkers to improve risk stratification. For example, coupling the CALLY index with radiomic features extracted from preoperative chest CT; tumor genomic alterations such as epidermal growth factor receptor, Kirsten rat sarcoma viral oncogene homolog, or tumor protein p53 mutations; or blood-based biomarkers such as circulating tumor DNA or IL-6 may enable the development of powerful prognostic algorithms. Such models could guide individualized adjuvant therapy and surveillance strategies (30).

Despite its strengths, this study has several limitations. To begin with, the single-center, retrospective design carries an inherent risk of selection bias and unmeasured confounding. Although we sought to mitigate these limitations through comprehensive multivariable Cox proportional hazards modeling, detailed covariate adjustment, propensity score matching, and additional stratified and interaction analyses, residual confounding cannot be completely excluded. Second, although our cohort is relatively large and reflects real-world surgical practice, the generalizability of the findings to other institutions, geographic regions, or non-surgical populations remains limited and requires external validation. Third, we evaluated the CALLY index at a single preoperative time point and did not assess longitudinal changes that may reflect dynamic physiological alterations during the perioperative period. Validation of the CALLY index through future multicenter studies involving diverse ethnic groups is needed, with special attention to elderly Western cohorts and individuals with multiple comorbidities, to refine its optimal cut-off values. Demonstrating its robustness across diverse settings will be essential for establishing the CALLY index as a globally applicable clinical indicator.

In conclusion, this study demonstrated that the CALLY index is an independent and consistent predictor of long-term OS in patients undergoing curative surgical resection for NSCLC, and that it provides incremental prognostic information beyond conventional clinicopathologic factors, despite only moderate discriminative performance as a single biomarker. Its prognostic performance surpassed that of other widely studied systemic inflammation-, nutrition-, and immunity-based indices, and remained significant in multivariable Cox models in the overall cohort, as well as after rigorous adjustment for baseline clinical heterogeneity through PSM. In addition, directionally consistent associations were observed in stage- and smoking status–stratified Cox models, with no evidence of effect modification in interaction tests. Importantly, the CALLY index is easily derived from routine laboratory data, making it highly practical and scalable for use in preoperative risk stratification. Although it did not predict RFS, its robust association with OS supports its role in assessing host-related vulnerability and guiding clinical decision-making. These findings suggest that the CALLY index may serve as a useful adjunct in preoperative evaluation to support more individualized care planning in NSCLC management.

## Data Availability

The raw data supporting the conclusions of this article will be made available by the authors, without undue reservation.
