# Supplementary material for: Prognostic value of the C-reactive protein-albumin-lymphocyte (CALLY) index in surgically treated non-small cell lung cancer
Source: Front Nutr. 2026 Mar 19;13:1784875. doi: 10.3389/fnut.2026.1784875 (PMC13043339; doi:10.3389/fnut.2026.1784875)
Supplement: Supplementary file 1 [file Table_1.docx]

| **Supplementary Table 1.** Formulas and unit conventions for the calculation of the CALLY index and other systemic inflammation–nutrition–immunity biomarkers used in this study. | |
| --- | --- |
| Biomarker | Calculation (unit conventions) |
| CALLY index | (Albumin [g/dL] × Lymphocyte count [/mm³]) / (CRP [mg/dL] × 10⁴) |
| NLR | Absolute neutrophil count (ANC) / Absolute lymphocyte count (ALC) (both in ×10⁹/L) |
| GPS | CRP >10 mg/L (1 point) + Albumin <35 g/L (1 point) → 0–2 scale |
| mGPS | CRP ≤10 mg/L → 0; CRP >10 mg/L & Albumin ≥35 g/L → 1; CRP >10 mg/L & Albumin <35 g/L → 2 |
| ALI | (BMI × Albumin [g/dL]) ÷ NLR |
| SII | Platelets × Neutrophils / Lymphocytes (all in ×10⁹/L) |
| HALP score | Hemoglobin (g/L) × Albumin (g/L) × Lymphocyte count (/L) ÷ Platelet count (/L) |
| AAPR | Albumin (g/L) ÷ Alkaline phosphatase (U/L) |
| PNI | 10 × Albumin (g/dL) + 0.005 × Lymphocyte count (/mm³) |
| PLR | Platelet count ÷ Absolute lymphocyte count (both in ×10⁹/L) |
| Abbreviations: AAPR, Albumin-to-Alkaline Phosphatase Ratio; ALI, Advanced Lung Cancer Inflammation Index; BMI, Body Mass Index; CALLY, C-reactive protein-Albumin-Lymphocyte; CRP, C-Reactive Protein; GPS, Glasgow Prognostic Score ; HALP, Hemoglobin, Albumin, Lymphocyte, and Platelet; mGPS, modified Glasgow Prognostic Score; NLR, Neutrophil-to-Lymphocyte Ratio; PLR, Platelet-to-Lymphocyte Ratio; PNI, Prognostic Nutritional Index; SII, Systemic Immune Inflammation Index. | |
